# Prime editor-mediated correction of a pathogenic mutation in purebred dogs

**DOI:** 10.1038/s41598-022-17200-4

**Published:** 2022-07-28

**Authors:** Dong Ern Kim, Ji Hye Lee, Kuk Bin Ji, Eun Ji Lee, Chuang Li, Hyun Ju Oh, Kang Sun Park, Seung Hoon Lee, Okjae Koo, Min Kyu Kim

**Affiliations:** 1 Laboratory of Animal Reproduction and Physiology, Division of Animal and Dairy Science, College of Agriculture and Life Science, Chungnam National University, Daejeon, 34134 Korea; 2 MK Biotech Inc., Daejeon, 34134 Korea; 3 National Institute of Animal Science, Wanju, 55365 Korea; 4 ToolGen Inc., Seoul, 08501 Korea

**Keywords:** Biotechnology, Molecular biology, Diseases

## Abstract

Canine hip dysplasia (HD) is a multifactorial disease caused by interactions between genetic and environmental factors. HD, which mainly occurs in medium- to large-sized dogs, is a disease that causes severe pain and requires surgical intervention. However, the procedure is not straight-forward, and the only way to ameliorate the situation is to exclude individual dogs with HD from breeding programs. Recently, prime editing (PE), a novel genome editing tool based on the CRISPR-Cas9 system, has been developed and validated in plants and mice. In this study, we successfully corrected a mutation related to HD in Labrador retriever dogs for the first time. We collected cells from a dog diagnosed with HD, corrected the mutation using PE, and generated mutation-corrected dogs by somatic cell nuclear transfer. The results indicate that PE technology can potentially be used as a platform to correct genetic defects in dogs.

## Introduction

Domestic dogs (*Canis lupus familiaris*) are the most variable mammalian species^1,2^. More than 400 breeds have been developed by intense artificial selection from a limited number of founders^1,3^. Consequently, purebred dogs have a greater risk of suffering from genetic disorders than any other species^4^. A number of scientific publications have described the health problems of purebred dogs^5–11^ and emphasized the need for action^9–14^; the problem has also been highlighted recently in public media^15^. As a result, many breeders are increasingly using DNA tests to reduce the frequency of deleterious mutations in their breeding programs^4^. However, no direct treatment has been developed to solve these inherent problems. In particular, canine hip dysplasia (HD) is the most common inherited polygenic orthopedic trait in dog; however, there is still no ideal medical or surgical treatment^16^.

Genome editing tools, such as CRISPR/Cas9 technology, can be a solution to this genetic problem. In particular, prime editing (PE), a novel and universal precision genome-editing technology has great potential for the correction of pathogenic alleles in purebred dogs. Unlike the conventional CRISPR/Cas9 system, PE does not induce double-strand breaks, which can induce random indel mutations at the target locus. PE was designed to generate a nick in single strands of the target genome locus, and then induce accurate target sequence switching using reverse transcriptase^17^. While PE was originally developed in human cells^17^, it has recently been used to develop genome-edited plant varieties and animals, including mice and fruit flies^18–20^. However, there is no report on the use of PE in dogs.

In this study, we used PE to determine whether point mutations causing canine HD can be corrected in canine fibroblasts. Furthermore, we attempted to confirm the possibility of producing genetically modified dogs by somatic cell nuclear transfer (SCNT) using PE-mediated genetically gene-corrected canine fibroblasts.

## Results

### Vector construction and prime editor guide RNA design

**Figure 1:**
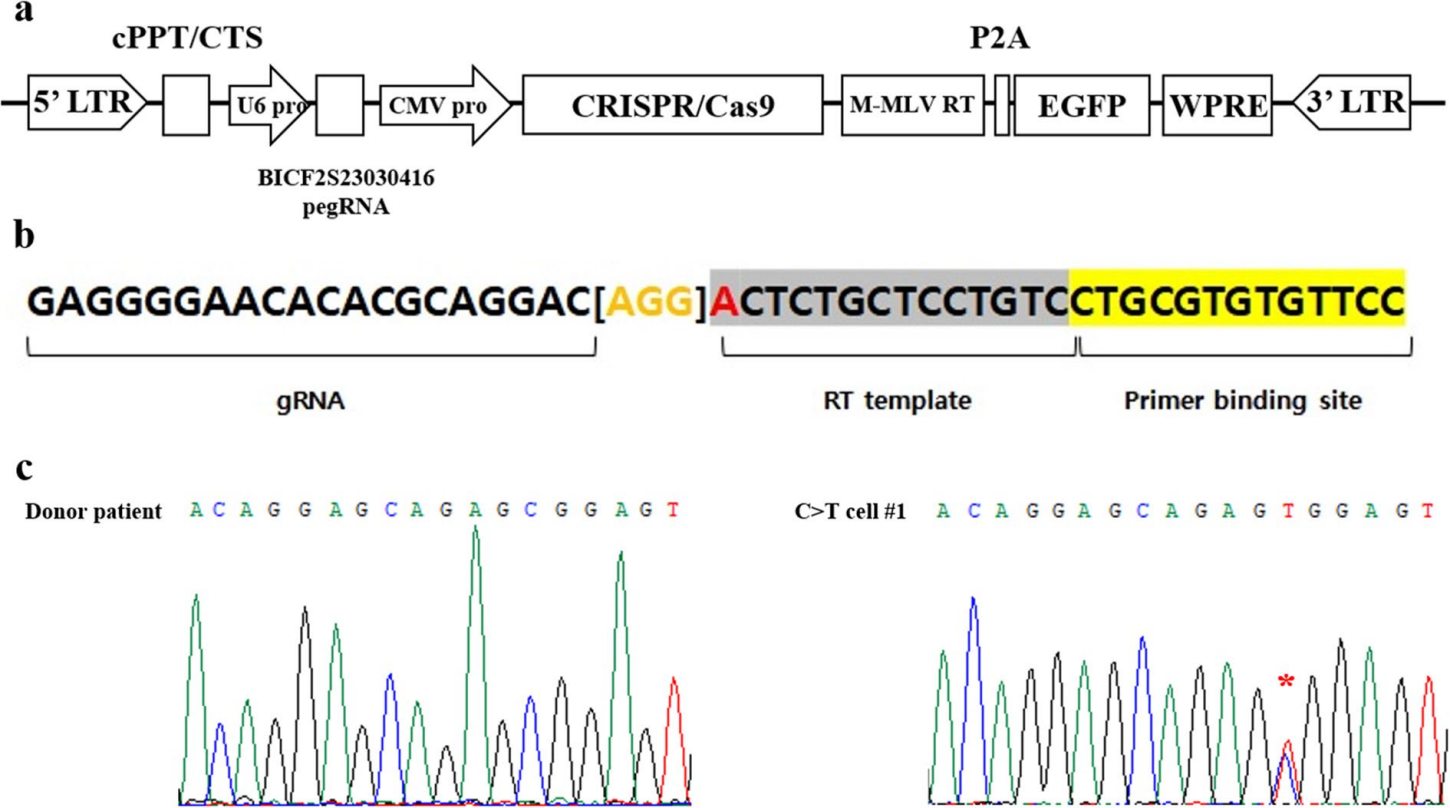
Correction of a point mutation in donor cells using prime editor (PE). (**a**) Schematic of PE vector. It consists of a prime editor that can correct SNP at the BICF2S23030416 locus and EGFP as a reporter. (**b**) Structure and design of PE guide RNA (pegRNA). The bracketed region in orange color indicates the scaffold for pegRNA^17^. The nucleotide (A) in red indicates the SNP mutation site. (**c**) Chromatographic analysis of the donor patient cells and C>T cell #1. The red asterisk indicates the target locus and confirms the C to T sequence correction mediated by PE.

We selected the target point mutation locus for PE-mediated gene correction based on our previous study that identified 25 SNPs correlating with hip dysplasia in dogs^21^. Among the 25 SNPs, BICF2S23030416 (Supplement Table 1) was selected as the genome editing target in this study because it showed the highest statistical significance for canine HD (*p* < 0.0001). The BICF2S23030416 SNP is located in an intergenic region on chromosome 4 and is hypothesized to function in regulating MSH homeobox 2 (MSX2). MSX2 has been utilized as a representative marker for cell ossification induction^22,23^. Our previous report showed that dogs (Labrador retrievers) with HD had a T to C point mutation at the BICF2S23030416 locus among the 25 SNP mutations assessed; therefore, we designed a PE vector to correct the T to C point mutation at this target locus. A lentiviral vector expressing both the PE enzyme (CRISPR/Cas9 nickase fused with M-MLV reverse transcriptase) and a prime editor guide RNA (pegRNA) was constructed and cloned (Fig. 1a). The pegRNA consisted of a primer binding site that could hybridize with sequences near the BICF2S23030416 locus, and a reverse transcriptase template containing the corrected genomic sequence at the point mutation site (Fig. 1b).

### Establishment of a gene-corrected canine fibroblast cell line

Ear fibroblasts were collected from a Labrador retriever dog diagnosed with HD (donor patient) and cultured in vitro. The T to C point mutation sequence at the BICF2S23030416 locus in the fibroblasts was determined by sequence analysis (Fig. 1c and Supplementary Figure 1, donor patient). Lentiviral particles, which is expressing PE, were transduced into the fibroblasts; the rate of transduction was measured by expression of the enhanced green fluorescent protein (EGFP) reporter gene. After 5 days of culture, gDNA was isolated from the transduced fibroblasts. Sequence analysis confirmed that the PE-treated fibroblasts had a T sequence at the BICF2S23030416 locus, mixed with C, indicating that PE successfully recovered one allele of the point mutation at the target site (Fig. 1c).

### Generation of gene-corrected dogs

**Figure 2:**
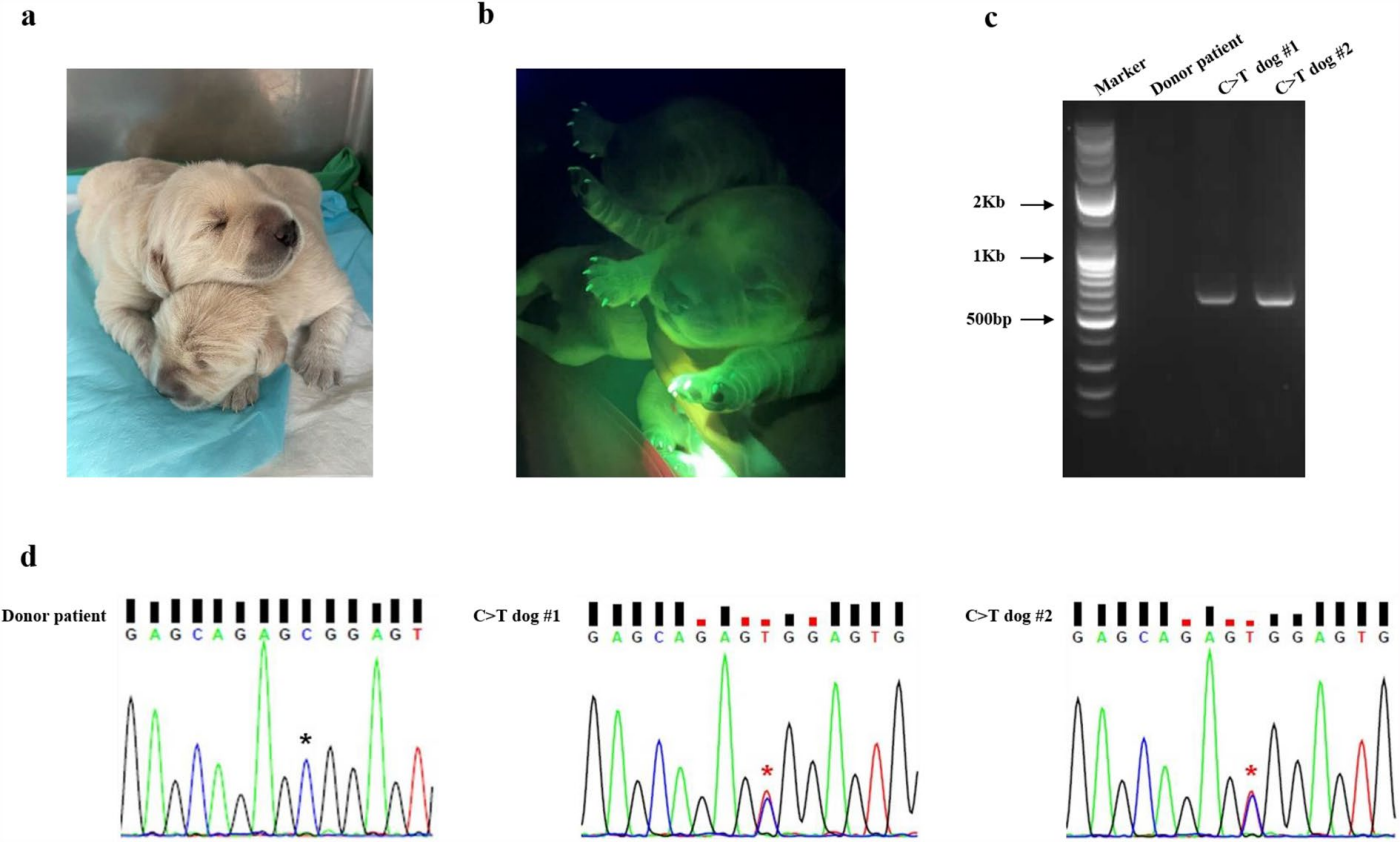
Generation and verification of gene-corrected dogs using somatic cell nuclear transfer (SCNT). (**a**) Bright field and (**b**) enhanced green fluorescent protein (EGFP) expression under UV light of the gene-corrected dogs. (**c**) PCR analysis of the vector construct integration in donor patient cells and gene-corrected dogs (C>T dog #1 and #2). Only the gene-corrected dogs showed a band at 600 bp. (**d**) Chromatographic analysis of target sequences from the donor patient (cells) and gene-corrected dogs. Blue star asterisks indicate SNP of donor patient and red star asterisks indicate corrected SNP sequence in C>T dogs #1 and #2.

After showing that we could correct the point mutation in fibroblasts, we demonstrated that we could produce gene-corrected dogs using SCNT. Among the fibroblasts produced using PE, we selected the ‘C>T cell #1′ for SCNT. In total, 18 reconstructed embryos were generated by SCNT using PE-treated fibroblasts and then surgically transferred into the oviducts of a surrogate mother (Table 1). Pregnancy was detected by ultrasonography at 40 days of gestation, and two puppies weighing 656 g (C>T dog #1) and 585 g (C>T dog #2 were delivered by cesarean section (Fig. 2a). We confirmed the integration of the PE vector by EGFP expression (Fig. 2b) and polymerase chain reaction (PCR) analysis (Fig. 2c). As expected, the C to T gene correction at the BICF2S23030416 locus was confirmed in both puppies (Fig. 2d and Supplementary Figure 1). These results are in line with the sequence analysis data from the PE-treated fibroblasts used as donor cells for SCNT (Fig. 1c). We also performed an in silico analysis of potential off-target loci from C>T dog #1 and C>T dog #2. No off-target mutations were identified in any of the analyzed loci (Table 2).

**Table 1:** Production of C > T dogs by somatic cell nuclear transfer (SCNT).

No. of transferred SCNT embryos	No. of recipients	No. of pregnancy (pregnancies/recipients, %)	No. of offspring (births/transferred embryos, %)
42	3	1 (33.3)	2 (4.7)

The number of oocytes used for SCNT is shown. A total of 42 mature oocytes were used. Three dogs donated oocytes, and one dog was used as a surrogate mother. A total of two cloned dogs were achieved.

**Table 2:** Sequencing analysis of potential off-target loci in gene-corrected dogs.

Off-target		Sequences	Ch	Position	Dir	Mismatches
gRNA		GAGGGGAACACACGCAGGACNGG	4	38226314	+	0
#1	Reference	GAGGGGAACACACG**G**AGGA**A**GGG	15	5224102	+	2
	C>T Dog #1	GAGGGGAACACACGGAGGAAGGG				
	C>T Dog #2	GAGGGGAACACACGGAGGAAGGG				
#2	Reference	GAGGGGAA**GT**CA**G**GCAGGACAGG	1	3887327	+	3
	C>T Dog #1	GAGGGGAAGTCAGGCAGGACAGG				
	C>T Dog #2	GAGGGGAAGTCAGGCAGGACAGG				
#3	Reference	G**G**GGGG**G**ACAC**C**CGCAGGACAGG	4	33314482	−	3
	C>T Dog #1	GGGGGGGACACCCGCAGGACAGG				
	C>T Dog #2	GGGGGGGACACCCGCAGGACAGG				
#4	Reference	GAGGGGA**C**CACA**A**GCAGGA**G**GGG	22	38923250	−	3
	C>T Dog #1	GAGGGGACCACAAGCAGGAGGGG				
	C>T Dog #2	GAGGGGACCACAAGCAGGAGGGG				
#5	Reference	GAGG**T**GAA**A**ACACGC**T**GGACAGG	31	38989958	+	3
	Donor patient	GAGGTGAAAACA**T**GCTGGACAGG				
	C>T Dog #1	GAGGTGAAAACA**T**GCTGGACAGG				
	C>T Dog #2	GAGGTGAAAACA**T**GCTGGACAGG				

Letters in bold indicate mismatches with the gRNA sequence. Ch: chromosome number; Dir: direction; Mismatch: number of mismatches.

## Discussion

In the present study, we successfully generated two gene-corrected dogs, cloned from a dog diagnosed with HD, using PE technology for the first time. HD is a musculoskeletal disorder caused by an unstable connection between the femoral head and acetabulum and is accompanied by severe pain. It is a common disorder in medium- to large-sized dogs, and is known to cause osteoarthritis, lameness and decreased mobility^24^. HD is a polymorphic disease caused by a combination of genetic and environmental factors^25^. Thus, to reduce the prevalence of HD, breeding strategies incorporating screening schemes are widely used^24^. However, studies that eliminate the cause of HD by directly controlling the causative gene have not yet been reported in dogs.

PE technology is effective and a potential solution for correcting genetic mutations in specific canine breeds. It is a simple and highly efficient gene correction system compared to that of CRISPR/Cas9-mediated homology directed repair (CRISPR-HDR)^17–19^. The CRISPR-HDR method is dependent on cell division events and requires an additional donor DNA template to correct genetic mutations. PE overcomes the shortcomings of CRISPR-HDR; it can be performed at any stage of the cell cycle and does not require additional donor DNA^17^. Therefore, PE is expected to be a very useful tool, enabling precise target sequence correction at specific loci in dogs. In addition, we also analyzed sequences from the potential off-target loci and did not find any unexpected mutations. The off-target analysis results revealed that the PE system is specific in canine cells. These findings are in line with previous studies demonstrating that the PE-mediated base conversion is highly specific^17,18^.

We corrected a single SNP from a dog with the HD phenotype. However, due to multiple SNP mutations are contributed to the HD, additional gene correction at the other SNP loci related to HD might be needed to generate a fully HD-recovered canine breed. We regarded the current study is the starting point to overcome HD of purebred dog. Thus, we integrated our PE vector into the genome of our gene corrected dogs and plan to perform further studies focused on correcting the additional SNPs. Integrated PE system will induce spontaneous nickase activity at the target site. In the current study, we did not find any indel mutation from our sequencing results; however, a more stable form of PE, such as RNP, can be recommended in further studies. Precise editing of pathogenic SNP in dog also provides valuable information for understanding the role of each SNP as it relates to HD. Since canine HD is remarkably similar in clinical expression and pathogenesis to that of human HD^26^, information gleaned from gene-corrected dogs may be very useful for understanding human HD. Thus, PE may be a very useful tool for generating genome-edited dog models to study human diseases.

In conclusion, we successfully confirmed the feasibility of PE in dogs and produced HD-related gene-corrected dogs using PE. To the best of our knowledge, this is the first study to adapt PE for use in a canine system. Further studies to analyze gait, behavior, and mobility of the current gene-corrected dogs, and the generation of additional gene-corrected dogs, are needed to understand the relationship between each SNP and HD.

## Materials and methods

### Ethics statement

The experimental procedures and methods used in this study were approved by the Animal Welfare and Ethics Office (2019012A-CNU-174), Chungnam National University, Daejeon, and performed according to “The Guide for the Care and Use of Laboratory Animals” published by IACUC of Chungnam National University. Female mixed dogs from 2 to 6 years of age were used in this study as oocyte donors and embryo transfer recipients. The dogs were housed indoors and fed once daily with water ad libitum. All methods are reported in accordance with ARRIVE guidelines (https://arriveguidelines.org) for the reporting of animal experiments in the Methods section.

### Construction of prime editor vector and production of lentiviral particles

The vector for PE was purchased from Addgene (Watertown, MA, USA: #135955) and modified to correct HD-related SNPs. Briefly, the CMV promoter was obtained by PCR using the primer sets 5′-gaattcttgacattgattattgactag-3′ and 5′-tctagaaatttcgataagccagtaagc-3′, and inserted into the vector by *EcoRI* and *XbaI* (NEB Inc., MA, USA: #R0101M and #R0145M) enzyme cuts. The pegRNA targeting the HD locus was newly synthesized and then added to the vector using *PacI* (NEB Inc., MA, USA: #R0547S) and *EcoRI*. Finally, the vector was confirmed through sequencing. The lentiviral particles of PE vector were produced by commercial vendor (Lugen SCI, Inc., Bucheon, South Korea).

### Collection and establishment of canine fibroblast cell lines, transduction, and transgene analysis

Fibroblasts were collected from the ears of an 18-month-old Labrador retriever diagnosed with HD (donor patient). The primary fibroblasts were cultured in vitro using culture medium composed of DMEM-GlutaMAX, 15% fetal bovine serum, and 1% penicillin/streptomycin solution (GIBCO, Inc.). For transduction, 100 multiplicity of infection (MOI) of the PE lentiviral particles, containing 1 μg/mL of polybrene, was transduced into 1 × 10^5^ fibroblasts per a well of 12-well plate. Transgene expression was confirmed by EGFP and integration of the vector was confirmed by sequence analysis.

### Collection of in vivo matured canine oocytes

We collected mature oocytes from dogs as described previously^27^. The concentration of progesterone in the blood was measured to optimize the hormone concentration for harvesting mature oocytes. After confirming the time of estrus, blood was collected, and progesterone was measured using VET Chroma (ANIVET Inc., Chuncheon, South Korea). When the analyzed progesterone level was in the range of 4–7 ng/mL we considered that day as ovulation. Three days after ovulation, mature oocytes were surgically collected. During the procedure, all dogs were treated with ketamine and xylazine at a concentration of 6 mg/Kg, and anesthesia was maintained with 2% isoflurane. After exposing the ovary and uterus, a 24G intravenous catheter was inserted into the oviductal lumen near the uterotubal junction, and the culture medium was flowed to collect mature oocytes. The culture medium was prepared by adding 2 mM NaHCO_3_, 1% penicillin/streptomycin, 0.5% bovine serum albumin, and 10% FBS to medium 199 containing 25 mM HEPES.

### SCNT and embryo transfer

For generating gene corrected dogs, SCNT followed by embryo transfer was performed following the method described elsewhere^27^. Briefly, in vivo matured oocytes with the first polar body were used for micromanipulation. Metaphase chromosomes were removed by aspiration from the oocytes. A single cell (C>T cell) was transferred into the perivitelline space of an enucleated oocyte, and each donor cell-cytoplast couplets were fused by two pulses of direct current (24–26 V for 15 μsec) using an Electro-Cell fusion apparatus. The fused SCNT embryos were chemical activated by incubating with 10 μM calcium ionophore (Sigma) and then 1.9 mM 6-dimethylaminopurine (6-DMAP). The activated SCNT embryos were surgically transferred into the oviducts of estrus-synchronized surrogates. Pregnancy was confirmed by ultrasonography at 30 days after embryo transfer.

### PCR validation and sequencing analysis

Transgene integration into the genome of transduced fibroblasts and gene-corrected dogs was confirmed by PCR. The PCR primers used to validate the Cas9 sequence in the vector were 5′-catcgctattaccatggtgat-3′ and 5′-ctcttgcagatagcagatcc-3′. These primer sets detected the linkage between the CMV promoter and dCas9 of the vector used in this study. Sequencing of the target locus was performed to validate the PE-mediated gene correction. The sequencing primers used were 5′-gacgccaagggagcagatatt-3′ and 5′-cctctcttatgagaacagcat-3′ (Bioneer Inc., Daejeon, South Korea). In addition, TA cloning was performed for accurate sequencing analysis using the products generated through PCR (Supplement Fig. 1). PCR products and T vector (Promega Inc., WI, USA: #A1360) were mixed at a ratio of 1:3, and DNA was isolated and purified from the bacteria colony generated by ligation (NEB Inc., MA, USA: #M0202) to extract DNA. After confirming the extracted DNA with *EcoRI* restriction enzyme, sequencing analysis was performed.

### Analysis of off-target mutations in gene-corrected dogs

Potential off-target loci were determined in silico using Cas-OFFinder (http://www.rgenome.net/cas-offinder/). We selected two potential off-target loci with two mismatches and another three loci with three mismatches compared to the genomic target sequences of pegRNA used in the study. The potential off-target loci were PCR amplified with genomic DNA from the C>T dog #1 and C>T dog #2 and sequencing analysis was performed (Supplementary Table 2).

### Ethics approval and consent to participate

In conducting this study, we collected cells from a retriever with hip dysplasia, and this was done after explaining the study to the retriever owner and consent was obtained. The experimental procedures and methods used in this study were approved by the Animal Welfare and Ethics Office (CNU-01090) of Chungnam National University, Daejeon, and performed according to the Guide for the Care and Use of Laboratory Animals published by the IACUC of Chungnam National University. All methods are reported in accordance with ARRIVE guidelines (https://arriveguidelines.org) for the reporting of animal experiments in the Methods section.

### Consent for publication

Not applicable.

## Supplementary Information

Supplementary Information 1.

Supplementary Information 2.

Supplementary Information 3.

## Data Availability

The datasets generated and/or analysed during the current study are not publicly available due to some data required for our further studies but are available from the corresponding author on reasonable request.

## References

[1] Ostrander EA (2019). Dog10K: An international sequencing effort to advance studies of canine domestication, phenotypes and health. Natl. Sci. Rev..

[2] Vilà C, Maldonado JE, Wayne RK (1999). Phylogenetic relationships, evolution, and genetic diversity of the domestic dog. J. Hered..

[3] Plassais J (2019). Whole genome sequencing of canids reveals genomic regions under selection and variants influencing morphology. Nat. Commun..

[4] Mellersh C (2012). DNA testing and domestic dogs. Mamm. Genome.

[5] Oberbauer A, Belanger J, Bellumori T, Bannasch D, Famula T (2015). Ten inherited disorders in purebred dogs by functional breed groupings. Canine Genet. Epidemiol..

[6] Nicholas FW, Wade CM, Williamson P (2009). Disorders in pedigree dogs: assembling the evidence. Vet. J..

[7] Nicholas FW, Crook A, Sargan DR (2011). Internet resources cataloguing inherited disorders in dogs. Vet. J..

[8] Ginja M, Silvestre A, Gonzalo-Orden J, Ferreira A (2010). Diagnosis, genetic control and preventive management of canine hip dysplasia: A review. Vet. J..

[9] Hedhammar Å (1999). European strategies to enhance canine genetic health. Eur. J. Companion Anim. Pract..

[10] Hedhammar Å (2005). Actions by FCI and WSAVA to promote canine genetic health. Eur. J. Companion Anim. Practice.

[11] McGreevy PD, Nicholas F (1999). Some practical solutions to welfare problems in dog breeding. ANIMAL WELFARE-POTTERS BAR-.

[12] Indrebø A (2005). Breeding healthy dogs–A breeder’s perspective. Eur. J. Companion Anim. Practice.

[13] Asher L, Diesel G, Summers JF, McGreevy PD, Collins LM (2009). Inherited defects in pedigree dogs. Part 1: Disorders related to breed standards. Vet. J..

[14] Summers JF, Diesel G, Asher L, McGreevy PD, Collins LM (2010). Inherited defects in pedigree dogs. Part 2: Disorders that are not related to breed standards. Vet. J..

[15] Hedhammar ÅA, Malm S, Bonnett B (2011). International and collaborative strategies to enhance genetic health in purebred dogs. Vet. J..

[16] Ginja M, Gaspar AR, Ginja C (2015). Emerging insights into the genetic basis of canine hip dysplasia. Vet. Med. Res. Rep..

[17] Anzalone AV (2019). Search-and-replace genome editing without double-strand breaks or donor DNA. Nature.

[18] Liu Y (2020). Efficient generation of mouse models with the prime editing system. Cell Discov..

[19] Jiang Y-Y (2020). Prime editing efficiently generates W542L and S621I double mutations in two ALS genes in maize. Genome Biol..

[20] Bosch JA, Birchak G, Perrimon N (2021). Precise genome engineering in Drosophila using prime editing. Proc. Natl. Acad. Sci..

[21] Choi, B. H., Kim, T. H., Lee, S. H. & Im, S. K. SNP for diagnosing hip dysplasia in dog and uses thereof. Republic of Korea patent 10-2012-0043793 (2013).

[22] Ichida F (2004). Reciprocal roles of MSX2 in regulation of osteoblast and adipocyte differentiation. J. Biol. Chem..

[23] Matsubara T (2008). BMP2 Regulates Osterix through Msx2 and Runx2 during Osteoblast Differentiation∗. J. Biol. Chem..

[24] Lewis TW, Blott SC, Woolliams JA (2010). Genetic evaluation of hip score in UK Labrador Retrievers. PLoS ONE.

[25] Wang S (2017). Genetic correlations of hip dysplasia scores for Golden retrievers and Labrador retrievers in France, Sweden and the UK. Vet. J..

[26] Zhou Z (2010). Differential genetic regulation of canine hip dysplasia and osteoarthritis. PLoS ONE.

[27] Lee JH (2016). Effect of acteoside as a cell protector to produce a cloned dog. PLoS ONE.

