# Author Correction: Prime editor-mediated correction of a pathogenic mutation in purebred dogs

**DOI:** 10.1038/s41598-022-20187-7

**Published:** 2022-09-21

**Authors:** Dong Ern Kim, Ji Hye Lee, Kuk Bin Ji, Eun Ji Lee, Chuang Li, Hyun Ju Oh, Kang Sun Park, Seung Hoon Lee, Okjae Koo, Min Kyu Kim

**Affiliations:** 1 Laboratory of Animal Reproduction and Physiology, Division of Animal and Dairy Science, College of Agriculture and Life Science, Chungnam National University, Daejeon, 34134 Korea; 2 MK Biotech Inc., Daejeon, 34134 Korea; 3 National Institute of Animal Science, Wanju, 55365 Korea; 4 ToolGen Inc., Seoul, 08501 Korea

Correction to: *Scientific Reports* 10.1038/s41598-022-17200-4, published online 28 July 2022

The original version of this Article contained an error in Figure 1.

As a result of an error during figure assembly, the C>T cell #1 panel in Figure 1c was a duplication of C>T dog #1 panel in Figure 2d. Figure 1 was replaced to show the correct data. The original Figure 1 is reproduced below for the record.

**Figure 1:**
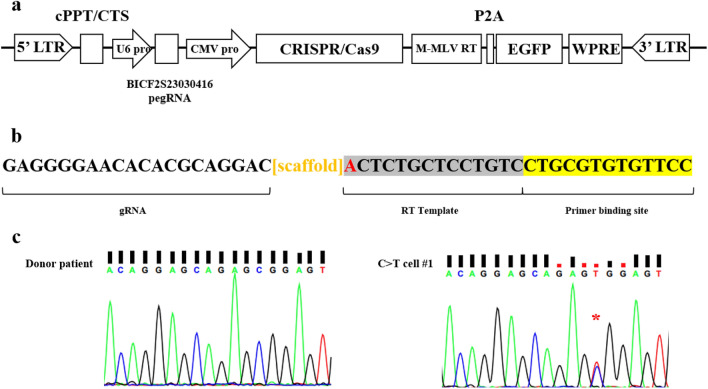
Correction of a point mutation in donor cells using prime editor (PE). (**a**) Schematic of PE vector. It consists of a prime editor that can correct SNP at the BICF2S23030416 locus and EGFP as a reporter. (**b**) Structure and design of PE guide RNA (pegRNA). The bracketed region in orange color indicates the scaffold for pegRNA^17^. The nucleotide (A) in red indicates the SNP mutation site. (**c**) Chromatographic analysis of the donor patient cells and C>T cell #1. The red asterisk indicates the target locus and confirms the C to T sequence correction mediated by PE.

The original Article has been corrected.

