# Supplementary material for: Prime editor-mediated correction of a pathogenic mutation in purebred dogs
Source: Sci Rep. 2022 Jul 28;12:12905. doi: 10.1038/s41598-022-17200-4 (PMC9334597; doi:10.1038/s41598-022-17200-4)
Supplement: Supplementary file 1 — Supplementary Information 1. [file 41598_2022_17200_MOESM1_ESM.docx]

**Supplementary Information**

**Prime editor-mediated correction of a pathogenic mutation in purebred dogs**

**Authors:** Dong Ern Kim^1^, Ji Hye Lee^1^, Kuk Bin Ji^1^, Eun Ji Lee^1^, Chuang Li^1^, Hyun Ju Oh^1^, Kang Sun Park^2^, Seung Hoon Lee^3^, Ok Jae Koo^4,*^ & Min Kyu Kim^1,2,*^


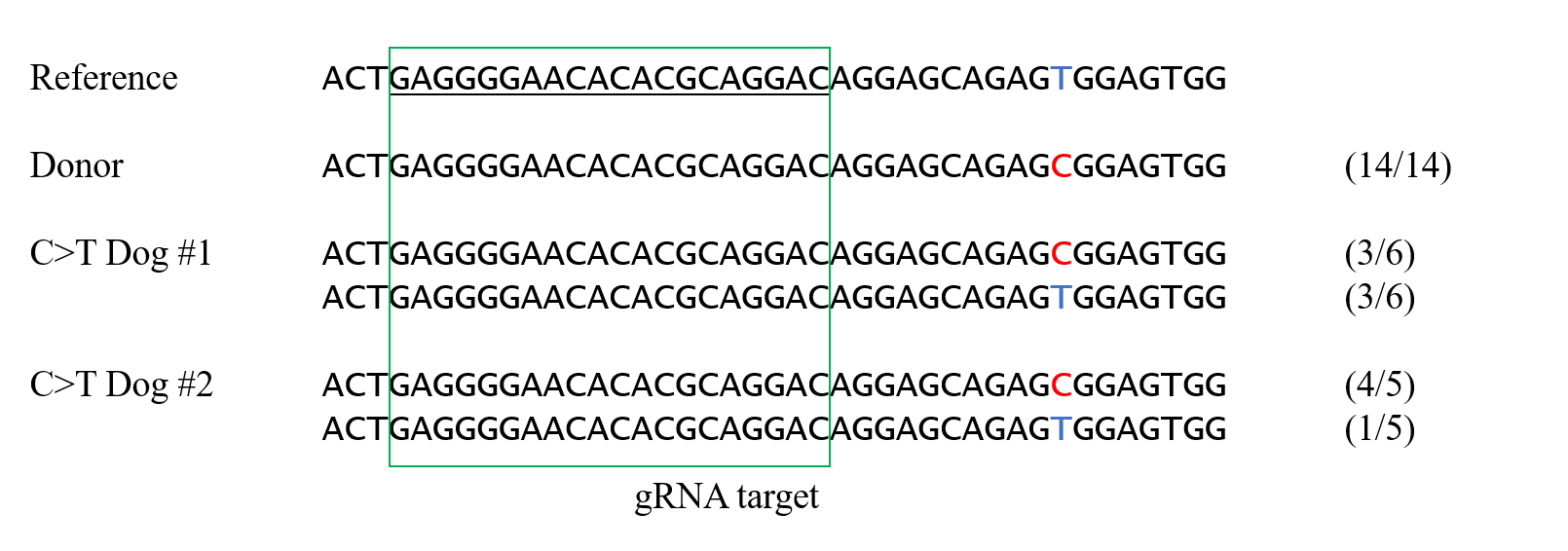


**Supplementary Figure. 1 Analysis of SNP mutations using TA cloning.** TA cloning was performed for point mutation analysis of SNP and sequencing analysis was performed. Donor is a retriever with HD, and C>T Dog#1 and C>T Dog#2 are cloned retrievers with point mutations by PE. C in red is the SNP shown in the donor with HD, and T in blue is the SNP induced by PE.

**Supplementary Table 1. List of SNPs associated with hip dysplasia in Labrador retrievers^1^.** In a previous study, significant levels of SNPs related to hip dysplasia in Labrador retrievers were summarized using statistical analysis. Among the 22,362 SNPs present in the genome, a total of 25 SNPs with significance for canine hip dysplasia were identified, including 11 on chromosome (CHR) 4, 4 on CHR 11, and 10 on CHR X (P≥0.01).

| **CHR** | **SNP** | **Position** | **Estimation** | **t-Stsatistics** | **Permutation_P** | **log(P)** |
| --- | --- | --- | --- | --- | --- | --- |
| 4 | BICF2P582065 | 21279946 | 8.544 | 4.507 | 0.00020 | 3.69897 |
| 4 | BICF2S23632496 | 23272314 | 7.510 | 4.167 | 0.00070 | 3.15496 |
| 4 | BICF2P95705 | 24055834 | 6.066 | 3.746 | 0.00030 | 3.52288 |
| 4 | BICF2P750308 | 24066370 | 6.066 | 3.746 | 0.00030 | 3.52288 |
| 4 | BICF2P783744 | 27241295 | 6.453 | 3.834 | 0.00050 | 3.30103 |
| 4 | BICF2P331356 | 27447828 | 11.300 | 4.608 | 0.00010 | 4.00004 |
| 4 | BICF2P1188097 | 27555277 | 6,257 | 3.748 | 0.00010 | 4.00004 |
| 4 | BICF2P676865 | 33248207 | 9.490 | 4.262 | 0.00030 | 3.52288 |
| 4 | BICF2S23030416 | 38226314 | 6.925 | 4.012 | 0.00010 | 4.00004 |
| 4 | BICF2G630166744 | 38258702 | 6.664 | 3.957 | 0.00020 | 3.69897 |
| 4 | BICF2S23110118 | 42752780 | 7.034 | 4.059 | 0.00020 | 3.69897 |
| 38 | BICF2S2373811 | 16185735 | -7.247 | -3.067 | 0.00040 | 3.39794 |
| 38 | BICF2P817345 | 16696343 | 5.764 | 3.895 | 0.00010 | 4.00004 |
| 38 | BICF2G63073656 | 16775306 | 5.025 | 3.415 | 0.00060 | 3.22192 |
| 38 | BICF2S23036049 | 18480476 | 4.989 | 3.274 | 0.00090 | 3.04581 |
| X | BICF2S2297550 | 91191649 | 6.197 | 3.527 | 0.00090 | 3.04581 |
| X | BICF2S22933756 | 99354996 | 7.311 | 4.011 | 0.00020 | 3.69897 |
| X | BICF2G6306331 | 108201633 | 7.500 | 3.623 | 0.00020 | 3.69897 |
| X | BICF2P227876 | 109116340 | 7.500 | 3.623 | 0.00020 | 3.69897 |
| X | BICF2G6305980 | 109652960 | 6.889 | 3.582 | 0.00050 | 3.30103 |
| X | BICF2P162522 | 111424321 | 7.367 | 4.106 | 0.00010 | 4.00004 |
| X | BICF2P593928 | 111997007 | 7.367 | 4.106 | 0.00010 | 4.00004 |
| X | BICF2S23044648 | 121338961 | -8.175 | -3.686 | 0.00050 | 3.30103 |
| X | BICF2P1430795 | 121460633 | -8.175 | -3.686 | 0.00050 | 3.30103 |
| X | BICF2S23222536 | 121643766 | 8.175 | 3.686 | 0.00050 | 3.30103 |

**Supplementary Table 2. List of primers used for off-target analysis.**

| **Chr** | **Name** | **Seq 5’ > 3’** | **TM** | **Size (bp)** |
| --- | --- | --- | --- | --- |
| 1 | cCH1_F | CTCAGGAGCAGTGAAACCGTC | 60 | 357 |
|  | cCH1_R | GTGGAGGTAGCCAAGTACCG | 60.2 |  |
| 4 | cCH4_F | GATCTTGTGCTGACAGCCTGG | 60.3 | 367 |
|  | cCH4_R | GCTCTGATGCCCCGAGATATT | 58.7 |  |
| 15 | cCH15_F | CAGGCAGAAACTGAGATTCTC | 55.4 | 437 |
|  | cCH15_R | CTCAGAGAATGTCAGAGGAGC | 56.6 |  |
| 22 | cCH22_F | GTTTCACCTAACTGCTAATGA | 52.6 | 432 |
|  | cCH22_R | GGAGCTTGAGTCAAAAAGATA | 52.6 |  |
| 31 | cCH31_F | GAAACGTACGGCTCAAAATGA | 56.3 | 409 |
|  | cCH31_R | GTTCAAACACGTGTCTCGTCT | 57.9 |  |

Chr; target chromosome
