# Supplementary material for: Prime editor-mediated correction of a pathogenic mutation in purebred dogs
Source: Sci Rep. 2022 Jul 28;12:12905. doi: 10.1038/s41598-022-17200-4 (PMC9334597; doi:10.1038/s41598-022-17200-4)
Supplement: Supplementary file 2 — Supplementary Information 2. [file 41598_2022_17200_MOESM2_ESM.pptx]

## Slide 1
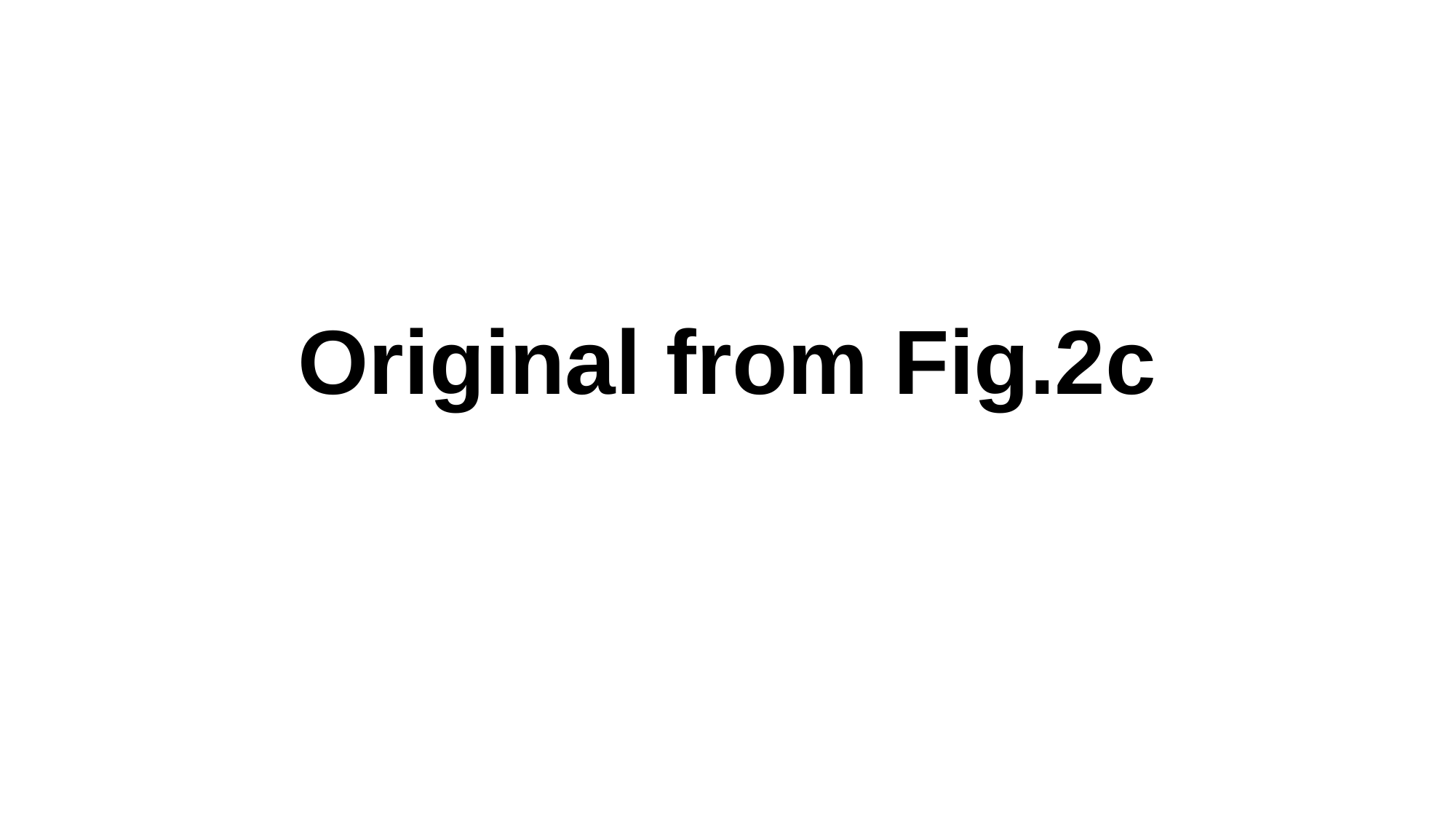

# Original from Fig.2c

## Slide 2
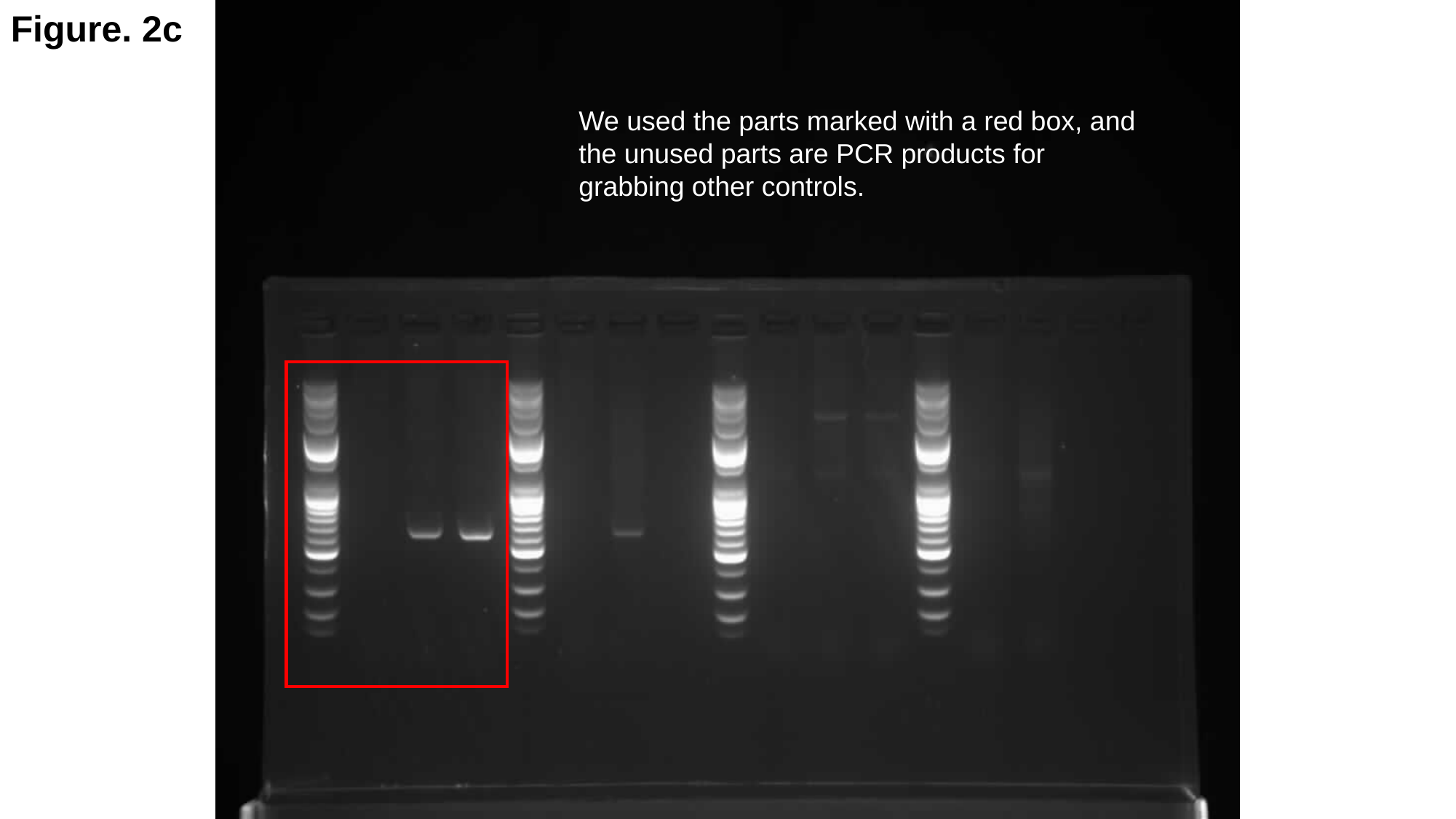

Figure. 2c
We used the parts marked with a red box, and the unused parts are PCR products for grabbing other controls.
