# Supplementary figures and images for: Prime editor-mediated correction of a pathogenic mutation in purebred dogs
Source: Sci Rep. 2022 Jul 28;12:12905. doi: 10.1038/s41598-022-17200-4 (PMC9334597; doi:10.1038/s41598-022-17200-4)

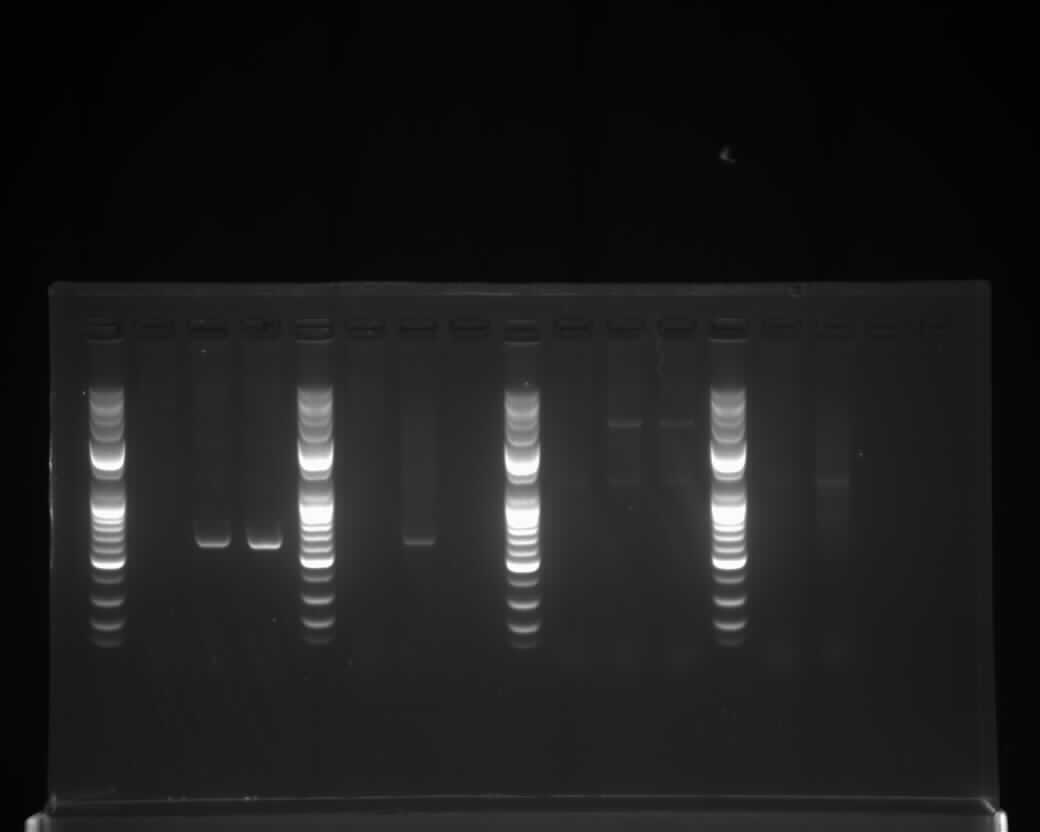

Supplement: Supplementary file 3 — Supplementary Information 3. [file 41598_2022_17200_MOESM3_ESM.jpg]
